# Society Proceedings

**Published:** 1890-01

**Authors:** 


### SOCIETY PROCEEDINGS.

PROCEEDINGS OF THE SOCIETY FOR THE STUDY OF COMPARATIVE PSYCOLOGY in connection with the Veterinary College, Montreal, Canada. The opening address of the year given by the Honorary President, Principal McEachran, was a most impressive one, and has been the means of greatly advancing the interests of various societies for the prevention of cruelty to animals. The following brief synopsis of the works of the society for the year is appended :—

I. A meeting of the Society for the Study of Comparative Psychology was held in the Veterinary College, the President, Prof. Wesley Mills, in the chair.

Mr. Dillon read a paper on the "Training of Young Dogs and Horses." The individual differences in both dogs and trainers has been established. All dogs and horses were not to be treated alike, but it was rarely, if ever, necessary to resort to severity. The disposition and intelligence of each must be studied in the application of general principles, and time and patience were essential to accomplish good and lasting results in all cases. He had noticed that certain horses, quiet in this country, had become vicious when taken to Britain, and could not account for it. One of the best preparations for the work of training was proper companionship with the animals to be rendered obedient. Firmness was to be combined with kindness. Mr. Dillon was opposed to inbreeding now so prevalent in the case of hunting dogs, as he was satisfied it lessened their intelligence, in some cases rendering them idiotic. He did not believe in training them before twelve months of age. In training to fetch and carry he used a ball of worsted, through which some wires had been pushed to ensure the dog taking it gently. It might be attached to a string. He also referred to the use of a cord attached to the dog. These were helps to be used judiciously, but not by rule of thumb. In answer to enquiries many points were brought out. A member from Montana stated that the training of the "wild horse" of the West was easy after he had lost the fear of man, and that real viciousness was rare. Mr. Dillon thought one cause of the "bucking" of many horses was the unnatural way in which they were strapped up.

Horses that were really vicious, the president thought, should be made to see that their actions punished themselves. This principle was employed by very successful trainers and was far more effective than punishment directly inflicted by the hand of the trainer in many cases. Evidence of fear on the part of the handler was pointed out as resulting in bad conduct by vicious animals that were under control by those that were firm and fearless.

A very able paper on "Reason and Instinct in Man and Animals," was read by Mr. Parker. He thought the so-called lower animals were different from us chiefly in degree of intelligence. In their instincts they were generally the superior of man and in reasoning not always his inferior. It was unfair to compare the man of culture and the mammals lower in the scale; but uneducated man or at least man proving but little of the results of training. He thought animals were "put out of count," so to speak, without a hearing, with the expression "O! they are mere brutes." One reason perhaps why so many took this view was that they were unaccustomed to analyze their own thinkings and actions, and so did not scrutinize closely those of the lower animals. It was important to do so, both to relieve man of his overweening conceit in his own powers and to prevent injustice to our fellow creatures of the dumb (to us) creation.

Mr. W. W. Robertson, present by invitation, agreed cordially with the views expressed by Mr. Parker.

The President thought the sort of education imparted a few years ago, generally on matters of the gravest concern, was often mixed up in high quarters with expressions which, in themselves, tended to perpetuate that indifference, ignorance and injustice to animals which is to be lamented. Why were children not taught in schools and in the family the duty of kindness to all things that breathe, as being related to us and not existing merely for us?

II. A meeting of the Society for the Study of Comparative Psychology was held in the Montreal Veterinary College, the president, Prof. Wesley Mills in the chair, the attendance of members being large. Mr. McWhinnie read a paper on "The Sympathetic Affection Between a Cow and a Pig." A pig five weeks old had been placed in a box-stall with a cow that had calved two weeks previously. Soon such good feeling sprung up between these two animals that when the cow was turned out into the yard the pig manifested great uneasiness, and finally refused to feed. On one occasion this pig was attacked by a dog, and ran into a ditch; the cow, being in a field near by, rushed to the pig's rescue, jumping the fence of the enclosure. The same writer instanced the case of a blind mare that had been provoked to kick by having tin pails tied to her tail, which led to the formation of the kicking habit. She was shod the first time with great difficulty, afterwards had corns cut, and on one occasion had come into the shop of her own accord and held up the affected foot, which was treated; the mare then being headed for home went thither without a halt.

Instances were cited of attachments existing between the dog and cat, pigeons and fowls, birds and cattle, horses and dogs, and even cats, and between many other kinds of animals. The president thought such cases illustrated the law of habit and the strength of the social instinct in animals. He instanced a case he had himself seen of a red squirrel that had been reared by a cat and when adult played about its foster mother like a kitten.

Prof. Baker mentioned the case of a fox terrier that sucked a litter of kittens alternately with their own mother. This was wise on the part of the terrier, for having lost her own young by death, the kittens relieved her distended teats. The president said it was undoubted that many animals associated relief from suffering, in an intelligent way, with its source. He had himself found that sick pigeons, even the wildest varieties, as carriers, when the treatment benefitted them, became tamer and were easily caught in a loft. Mr. Wieland read a paper on the nature of the intelligence of a mongrel dog (bred from a bull-dog and spaniel) he had himself possessed. Among the most interesting points that seemed to have been established by this observer was the existence of the ability in this dog to distinguish between words closely related in sound as 'cat' and 'rat.' Though puzzled at first the dog soon learned the difference. The general intelligence of this dog was high. The President thought that the mongrel often excelled the pure breeds in general intelligence. The great difference was that some of the breeds, in fact most of them, were specialists, and in certain directions they could be taught more readily and attain higher results. An interesting communication was read from a former member, in which, among other things, the intelligence of a horse, in turning on and off the water from a faucet, was described. Mr. Parker instanced the case of a colt that swam a lake instead of taking the long road around it, to reach her home. This communication led to many others bearing on the fact that animals of various kinds can return to their homes over great distances, often by ways they have never travelled.

III. A meeting of the Society for the Study of Comparative Psychology was held in the Montreal Veterinary College, the president, Prof. T. Wesley Mills, in the chair. A student of human medicine of the second year related an instance of apparent heredity of a mental trait. A mare and seven of her colts, though not broken by the same person, showed the peculiarity of stopping with extreme suddenness at the word "whoa." A member of the society had known in Ireland a stallion that always, when going through a stable doorway, etc., made a sudden rush, lowering his head at the same time. This behavior was also evinced by his offspring to the fourth generation. It seemed to have originated from this stallion having been struck when passing out on one occasion, he then having hurt himself against the side of the passage. Another member stated that he had owned a horse perfectly gentle with ladies, and when driven by a man that was intoxicated, even stopping if the sleigh was overturned and looking around, but comparatively unmanageable when driven by men if sober. Such an animal must be credited with much intelligence. The president pointed to the sense of humor of horses and their tantalizing behaviour at times, as when in a pasture field. One member had known of a large horse that would allow the owner's wife to feed him, but not himself. He on one occasion as an experiment put on one of his wife's dresses, but the horse detected the imposture and behaved as usual.

Gun-shyness in dogs was discussed. It was found that the same principle was illustrated in horses. One of the members had worked a horse that was greatly terrified by the sound of the whistle of locomotives, and on one occasion he had been held while the whistle was blowing, and after a violent trembling had fallen dead. Reference was made to the conduct of the collie dog of an absent member. When the latter was packing his trunk he could not be induced to leave the spot and contrary to his usual custom had insisted upon following him everywhere through the house. He had seen the same trunk packed for his master once before and moped during his entire absence. His human like behaviour could only be explained on the same principles as apply to our own minds. It seemed but right to take such a dog on the journey, and this was done.

The president said the realization of the fact that something very grave was to happen could scarcely have been shown more fully by a human being than in the case of a dog of unusual intelligence. He once had occasion to send off by express, when placed on the box that was used to transport him, it was very painful to witness the evidence of his deep emotion. A member from the west instanced the case of a collie that after the death of the herder had safely driven into the corral the 7,000 sheep that composed the herd. This same dog during a blizzard had remained out all night looking after the strayed animals. Another member related how a gentleman living at Point St. Charles seeing a rat in a bad plight had given the creature milk and continued to do so as it appeared day after day in his yard. It became tame like a kitten and played around among his children, but never entered his kitchen. Regret was expressed that a small portion of the press of the city saw fit to publish occasionally a series of narratives of animals, especially of encounters between them and men, which to say the least were very highly colored and not calculated to advance the cause of comparative psychology or truth in any form.

IV. The fifth meeting of the season of the Society for the Study of Comparative Psychology was held in the Veterinary College, with the president, Dr. Mills, in the chair, the attendance of members being large. Mr. Austin read a paper on Ants, based on a study of their habits on the prairies of Colorado. Their methods of surmounting obstacles, building their nests, etc., were described. One species of large size built a nest as large as a bushel basket. The essayist had experimented by catching centipedes and leaving them tied within reach of the ants. Their behavior under the circumstances pointed to the possession of intelligence of no ordinary degree. Mr. Austin thought that if the size of the two animals were considered, it was doubtful whether, from this point of view, that of man himself equalled the intelligence of the ant. Humorous reference was made to the "dry wash" accomplished by the ants for the Indians and others, where water was scarce.

After remarks by the president and others, Mr. Simpson read his paper on the intelligence of the pig and the goose. He had noticed that a little pig among some larger ones did not get its full share of the buttermilk served out, so he had supplied it with some before the others were admitted. The pig soon learned to come of it's own accord, and since it came only on buttermilk days, it evidently had powers of discrimination. It varied within but a few minutes n coming for its milk on the days that this was served out to the hogs. He also had two very small pigs that after being kept for two weeks went home, when free, a distance of two miles. Mr. York also bore witness to the capacity for education and the appreciation of kindness by pigs. The president thought the hog a much underestimated animal.

Mr. Simpson then treated the psychology of geese, referring to their concerted action in a flock, the caution when feeding of the wild species, etc. In the Southern States geese had been trained to pick weeds and leave the cotton plant untouched.

The president stated that he was now engaged in a study of bird psychology by direct observations.

After further discussion, the meeting adjourned.

---

THE ILLINOIS STATE VETERINARY MEDICAL ASSOCIATION held its Seventh Annual Meeting at the Sherman House, Chicago, November 6th and 7th.

Called to order at 2 P. M., Wednesday the 6th, President W. L. Williams in the chair.

After roll-call and reading of the minutes the president read his annual address. After touching upon various topics of interest to the welfare of the society, and paying tribute to the memory of his former business partner, Dr. Jas. Brodie, deceased. Dr. Williams spoke at length upon the subject of Veterinary Journalism. An effort had been made to start a veterinary journal for the West, at Ames, Ia.

At the election of officers Dr. Williams was elected to his third term of office as President. The other offices were filled as follows:

1st Vice President, R. W. Story, Princeton; 2d Vice President, Jas. Bond, Streator; 3d Vice President, J. F. Reid, Decatur; Recording Secretary, J. F. Pease, Quincy, second term; Corresponding Secretary, C. E. Hollingsworth, La Salle; Treasurer, J. T. Nathens, Delaware; Censors, Drs. Casewell, Baker and Redner, of Chicago.

The names of S. H. Kingery, Chicago, '88; W. F. Weese, Ontario, '87; E. W. Lawton, Chicago, '89, were placed on the membership role by acclamation.

A. S. Alexander of the *Farmers' Review*, Chicago, was elected to *honorary membership*.

The Corresponding Secretary handed in the report of the Treasurer, and in his own report, read a communication from the Indiana Association inviting our members to attend their next meeting at Terre Haute, Indiana. The invitation was accepted with a vote of thanks and ordered placed in the records.

Correspondence from Dr. A. Liautard was also read, regretting his inability to attend and address the meeting.

Dr. Story, of Princeton, read an interesting paper on Azoturea. He followed the *etiology* and *diagnosis* of the disease according to the latest authorities and personal observation, and for treatment recommended quietude, *venesections*, a *cathartic*, and sedatives, with stimulating applications to the loins and early and frequent use of the catheter.

*Discussion:*—Prof. Baker asks for the death rate with this treatment. *Answer.*—About *one* in *fifteen*.

Prof. Baker's cases in this city, are more severe; about 90 per cent. become delirious, anæsthetics are necessary; about 40 per cent. are fatal.

He thinks cathartics are contra-indicated. Believes delirium due to uræmic poisoning. Gives Gelseminum and Bromide of Potassium. Gets marked *diuresis* without danger to kidneys.

Dr. Williams avoids cathartics; depends on laxations as oil, etc., frequent use of the catheter, and plenty of medical non-interference.

Dr. Hollingsworth, of La Salle, read a carefully prepared paper on *Enteritis.* He believes in opium, or morphia hypodermically, hot fomentations and warm enemas, with sedatives in full doses internally.

*Discussion:*—Dr. Williams quoted Walley on the theory of tension of the bowel being the cause of the rapidly fatal form of the disease.

The essayist has not found this in his P. M.'s. Prof. Baker finds an occasional case of tension, but many careful P. M.'s. fail to reveal any.

He finds numerous *Strongyli tetracanthi.* Dr. Williams bleeds now, in all plethoric cases of the so-called apoplectic variety.

Dr. Withers coincides, but finds the cases in the city are *not* plethoric as a rule.

Then followed a discussion on varieties of trocars and the manner of using.

Abscesses are caused by dirty trocars and by illy-constructed ones carrying hairs in with them.

The association adjourned to attend the annual banquet.

The meeting re-convened at 2 P. M., Thursday the 7th, to listan to an interesting parer by Dr. Hirnbauch, of Lafayette, Ind., on "Diathesis and Idiosyncrasy." The paper was not discussed.

The standing committee's were appointed, and three committees failing to report were discharged. The committee on legislation reported through their chairman, Dr. Casewell. The funds of the special assessment were fully accounted for, and the balance turned over to the treasurer. The doctor reported that strenuous efforts had been made to have the bill pass, and he, as well as Pres. W. L. Williams and Prof. Baker had appeared before the legislative committee to urge its passage. The original bill had been made as strong as dared, but had been changed by the Committee on Dairying, etc. It had failed; partly by reason of an antipathy in the last legislature towards *boards and commissioners*, but most by opposition on the part of younger men of the profession, both *in* and *out* of the association, who thought the bill not strong enough. This the doctors censured highly, and proposed that the next committee be composed of young men, as *they* and not the older ones with well established practices would be benefitted by a bill. This report was accepted as the report of the committee, and ordered, together with the corrected bill, spread upon the minutes.

Then followed a paper from Dr. Lanigan, of Wenona, on the "Principles of Feeding." As the writer was unavoidably absent the paper was read by the corresponding secretary and was not discussed.

A paper by Dr. J. T. Nathers, on the subject of "Parturient Appoplexy" brought out a long discussion on the pathology of the disease.

Brof. Baker consideres the term *apoplexy* misleading, it must be an anæmia of the nervous system. Prof. Hinebach agreed with him. His treatment is Pilocarpin 15 to 20 gr.; Eserin 1 to 1½ gr. not repeated.

Cold to head and pack to the body. *Question* by Williams: Why do you apply cold to the head for anæmia of the brain? The professor acknowledged it was inconsistent with the theory, and he did not see that it did any good. Objected by Dr. Pease, that these causes are always plethoric; again, is not dilatation of the pupil a sign of hyperæmia? *Answer.*—It is also found in anæmia of the brain. Profs. Baker and Withers hold that the disease is due to a lack of use of the nerve-energy naturally stored up in the nerve cells for the act of parturition. The animal dies from coma and collapse. Pilocarpine and Eserin are both powerful nerve stimulants. Dr. Williams admits the *use* of stimulants, but finds it impossible to safely administer them by the mouth. He employs Pilocarpine with ice bag to poll and cold wet pack to body.

The association then adopted resolutions of condolence in the loss of Dr. James Brodie, formerly a member, after voting thanks to the visitors and retiring officers, as also to "our host." The association adjourned to meet in Peoria during February next.

J. F. PEASE,

QUINCY, ILL.

*Recording Secretary.*

---

*The New Jersey State Veterinary Society* held a *special* meeting at Sanger Hall, Newark, N. J., on Tuesday, Oct. 29, 1889.

The President, Dr. E. L. Loblein, called the meeting to order at 7 o'clock P. M.

The roll call was answered by Dr. J. C. Corliss, Dr. I. N. Krowl, Dr. C. Kuehne, Dr. E. L. Loblein, Dr. M. H. Lowe, Dr. M. E. Mattison, Dr. E. R. Mercer, Dr. J. Naylor, Dr. L. R. Sattler, Dr. A. Sherk, Dr. A. Vogt, Dr. E. R. Voorhees, Dr. W. M. Mook, Dr. A. H. McIntosh, and Dr. J. Hopkins. Mr. E. D. Bachman and Mr. C. L. Thudichum, students of veterinary medicine at the American Veterinary College were also present.

The Secretary's report was adopted as read.

The Secretary read the call for the meeting as follows:—

Dr. E. L. LOBLEIN,
*President of the New Jersey State Veterinary Society.*

SIR:—We, the undersigned members of the New Jersey State Veterinary Society, respectfully request that you call a *special* meeting of the Society at an early date for the transaction of business under the new law entitled "An Act to protect the title of Veterinary Surgeon and to regulate the practice of Veterinary Medicine and Surgery in New Jersey." And such other business as may come before this meeting.

(Signed) M. H. LOWE, D.V.S.
J. F. AUTENREITH, D.V.S.
JAMES MCCAFFREY. D.V.S.
E. R. VOORHEES, D.V.S.
L. R. SATTLER, V.S.

The first business in order was for the election of a counsel to the Society, and Dr. Wm. H. Lowe presented the name of the Hon. R. Wayne Parker, of Newark, N. J.; he was accepted by a unanimous vote.

Dr. Corliss moved that the committee which had been appointed at the last meeting be increased to five instead of three as at present; it was seconded, and when put to a vote it was carried. The President appointed Dr. Voorhees and Dr. Hopkins to act with Drs. Lowe, Sattler and Corliss, who had been appointed at the last meeting; this committee to be known as the "Committee of Jurisprudence." That they confer with our counsellor, and are appointed with power. The committee drew up the following resolutions which were unanimously adopted:

WHEREAS, The Legislature of the State of New Jersey passed an act entitled "An Act to protect the title of Veterinary Medicine and Surgery in New Jersey," Chapter XXIV., Laws of New Jersey, 1889, approved March 4, 1889; and

WHEREAS, Said law requires all practitioners of Veterinary Medicine and Surgery who are graduates of a legally chartered veterinary college to register in a book kept for that purpose, in the office of the county clerk in the county in which they reside; and it is made the duty of county clerks to keep a record of each diploma together with affidavit of veterinarian that he is the person to whom the diploma was issued, etc.; and

WHEREAS, Said law also requires all persons who have assumed the title of veterinary surgeon or analagous title, in this State for five years preceding the passage of this law, without being entitled to the degree of veterinary surgeon, shall be allowed to continue the use of the title; bnt such persons shall appear before the county clerk of the county in which they reside, within six months after the passage of this act (March 4, 1889), and make affidavit to that fact; and the county clerk is then required to register such persons as "existing practitioners" Now, therefore, be it

*Resolved*, That realizing the importance and monetary value of our stock industry, and appreciating the grave danger of communicable diseases from domestic animals to man through our food supply, and the intimate relations existing between man and animals (horses, dogs, etc.), we gladly commend the wisdom of our legislators in the enactment of a law designed to afford protection to owners of domestic animals against impositions practiced by charlatans and empirics who have *assumed* the name and functions of an honorable profession.

*Resolved*, That we respectfully demand of those in authority that chapter XXIV., Laws of 1889, shall be strictly enforced, and especially call upon the prosecuting attorneys of the different counties to give particular attention to the prosecution of offenders, and in order that every effort shall be made to enforce the law, we hereby authorize our counsel, Hon. R. Wayne Parker, of Newark, to take such measures as in his judgment are necessary to enforce the law.

*Resolved*, That all veterinarians of New Jersey, graduates of legally chartered veterinary colleges, be requested to send their names to our Secretary, Dr. Charles Keuhue, Jersey City, N. J., and be enrolled as members of this association, and also to forward full information as to infringements of this law (ChapterXXIV.) in their respective couuties, in order that they may receive the attention of our attorney.

After transactiong other routine business the meeting was adjourned.

CHAS. KUEHNE, D.V.S.,
*Secretary.*

*The regular meeting of the New Jersey Veterinary Medical Association* was held in Saenger Hall, Newark, N. J., on Thursday, December 12th. Meeting called to order by President Dr. J. W. Hawk of Newark. Roll was called and a good number of the members answered. Minutes of last meeting approved. Col. E. S. Edwards, I. C. Kilburn, Franklin Dye, Secretary of the State Board of Agriculture, Dr. J. H. Hammill, late of New York College, and others were present for the first time. The president opened the meeting with a good lively address in which he spoke of the good condition the association was in. Many new members had been enrolled, and they were, it was said, of a character to add weight and dignity to the profession. Complaints had been heard from various districts showing that contagious diseases have not been entirely eradicated. Pleuro-pneumonia to a degree in certain sections. Letters from Dr. D. E. Salmon, Chief of the Bureau, Animal Industry of the United States, Hon. J. M. Rusk and others were read. An interesting discussion ensued on the bill requiring registration of veterinary surgeons. Dr. Hammill believed in it so far as it went, but claims that it did not go far enough. He thought the profession should have placed a higher estimation upon its character and services than to limit the price of registration to $1. The price of registration should have been fixed at $10. This would have added dignity to the cause. Dr. Miller, of Camden, spoke of the difficulty of getting a bill of any kind through the Legislature, and the association had accepted for the present what they could get on the principle that the half loaf is better than no bread. Franklin Dye, of this city, stated that there is now need of an efficient corps of veterinarians in New Jersey. The horses of this State are valued higher than those of any other State in the Union, and the same is true of the milk cows and beef cattle. We cannot afford to risk the lives of this valuable stock. Concerning the State Veterinary Association, the State Board of Health, the Bureau of Animal Industry and the State Board of Agriculture, they are working on too many divergent lines. They should be brought into closer sympathy of understanding and action as to the sphere and work of each. A vast amount of money is expended for the extermination of disease which does not seem to give corresponding results. Diseases of cattle especially are assuming new complications and are becoming serious. They affect not only the pocket of the owner but also the consumers of milk, and this is true of diseased pork. This whole subject is one of general interest, and may require further Congressional and State legislation.

An interesting paper on "Swine Plague" was read by Dr. Julius Gerth, Jr., stating that to-day this disease can be found in every State of the Union.

An animated and extended discussion arose over the proper diagnosis of the disease which has been termed by some veterinarians *Bovine variola*, or an aggravated form of cowpox, caused by the buffalo fly; by others a new disease not yet accounted for. Dr. Dunston, of Morristown, said he believed that the milk from these cows is being sold to the public. Dr. Higgins, of New Brunswick, said that he knew that milk from cows suffering from this disease was being sold in the community.

The President was instructed to appoint a committee of five to investigate and prosecute all cases where veterinarians are practicing without being registered.

A committee of three, consisting of Dr. W. B. Miller, of Camden, Dr. Julius Gerth, Jr.. of Newark, and Dr. W. H. Cooper, of Trenton, was appointed to attend the next annual meeting of the Board of Agriculture.

Yours respectfully,

W. H. COOPER, *Secretary.*

---

*Keystone Veterinary Medical Association.*—The regular monthly meeting was held in Philadelphia, Nov. 2, 1889, in the Veterinary Deparrtment of University of Pennsylvania, Dr. Huidekoper, President, in the chair.

The Secretary being absent, reading of the minutes was postponed. Under new business Dr. Zuill moved, seconded by Dr. Rayner, that all by-laws in relation to fines be expunged from the book, and that all fines that have accumulated against members shall be cancelled. Dr. Harger proposed for membership, C. H. Magill aud W. B. Werntz, graduates of the Veterinary Department of the University of Pennsylvania. Dr. Hoskins read a paper on osteo-porosis. Remarks were made by Drs. Zuill, Goenter, Kooker, Huidekoper and others. Dr. Hoskins reported a case where the autopsy revealed an abscess attached to kidneys, large, and filled with fœtid pus. Dr. Zuill reported case of pelvic abscess with recovery, but the animal always had a roach back, lived one year, when he died of pelvic abscess. Dr. Zuill also reported another case of internal abscesss; he thought all these cases due to strangles.

Dr. Felton, the Secretary, now appeared with the minutes of last meeting. It was moved and seconded to take up the regular order of business. Dr. Felton read the minutes of the last meeting, which were approved. The Treasurer made his report: total expenditures for the year, $109.28, with a balance in the treasury of $4.49. Report received, and referred to board of trustees. Dr. Cullen reported a case of obstruction of bowel remarkable for the size and great rotundity of the calculus.

The Keystone Veterinary Medical Association has 29 members in good standing, with 4 applicants for next meeting, and a loss of one member during the past year. Officers for current year:—President, Dr. Huidekoper; Vice President, W. H. Hoskins; Secretary, W. H. Ridge; Treasurer, Chas. T. Goentner; Board of Trustees. Drs. Hoskins, Zuill, Glass, Eves and Hartman. There has been a great deal of work done during the past year by this association; The association is exact in regard to choice of members, but extend a welcome invitation to the profession to visit them and join in the debates. The association has nearly doubled its number of members within the last two years, but there are many graduates in the near vicinity, who do not belong to it.

The monthly meeting of the Keystone Veterinary Medical Association met at Philadelphia, December 7, 1889, at its usual place. Dr. Huidekoper, President, being absent, Vice-President Dr. Hoskins presided. At roll-call 14 members responded. The minutes of last meeting were read and approved. There not being a quorum of trustees present, the Vice-President appointed

Drs. Thos. B. Rayner, W. S. Kooker, S. J. J.Harger, *pro tem.* to fill vacancies. It was moved that an intermission of fifteen minutes be taken while the board of trustees held their meeting. C. H. Magill and W. B. Werntz wishing to become members appeared before the board. The meeting again came to order. The board of trustees reported favorably, and both candidates were elected active members. Committee on Legislature reported; Dr. Kooker explained the proceedings in the Lancaster County courts; Dr. Hoskins called attention to the number of apparent false registries in the city, beside many false post regtistries. There has been no court decision rendered in regard to registration.

The alteration of the by-laws was next taken up; Dr. Kooker moved that Article IV., Section V,, and Article VIII. be stricken off the the by-laws. This was seconded by Dr. Ridge and carried by a unanimous vote.

Dr. Kooker read a communication from the interstate association, a new organization. He also read a resolution that the place of meeting of the Keystone Veterinary Medical Association be changed from the University of Pennsylvania to a more central location, signed by Dr. Hoskins and Kooker.

Dr. Lintz read a paper on Influenza, explaining it in full. Remarks were made by Dr. Isaiah Mitchener, a visitor who has been in active practice of veterinary surgery for over fifty-three years; the doctor cited an epizootic of influenza in 1850, when it was very fatal, one man losing 100 horses in the city of Philadelphia. The treatment then prescribed was to bleed and physic, which made short work of them. He said it was a specific disease. He found that stimulants were best suited to cure it. He gave

℞ Ammoniæ carb.
Gentianæ pulv. āā ℥ij.
Camphoræ ʒj.
M. ft. Pulv. et chart no iij.
Sig. One three times a day.

and said that the epizootic of 1872 was another disease, due to a highly oxygenized condition of the atmosphere, a wave of such air passing over a territory robs the blood of a portion of its iron, producing an epizootic which was not so formidable a disease as influenza. This theory was freely discussed by Drs. Lintz, Kooker, Bridge, Hoskins, Werntz and others. Dr. Werntz asked why all kinds of animals were not acted upon alike? Which was answered by Mr. Ridge by saying some animals had immunity, which was something no one could explain. Dr. Kooker was asked if he thought the epizootic of 1872 was influenza. He replied in the negative. Dr. Felton reported a case of tetanus due to being chilled off suddenly after exercise, which took the form of opisthiotonis with some trisimus. He gave chloral and belladona which seemed to relieve him. As he began to improve slowly the mnscles of posterior part first became relaxed, then the muscles of back until now only the muscles of head are affected. The obliques of head are hard, yet otherwise he has made complete recovery. There was no injury received to produce the symptoms.

Dr. Isaiah Mitchener reported two cases within the last year similar to Dr. Felton's case. Dr. Williams reported a case of laminitis in which only one fore foot was involved, which caused tetanus. Autopsy proved founder in only one foot. Dr. Hoskins cited a case of founder in three feet. Dr. Kooker reported a case of tetanus in a mule attributed to feed of corn, which he cured by giving a large dose of aloes, then giving hot water to drink. The water was so hot that he could not bear his hand in it, which the mule drank with relish and recovered. This mule on being fed corn again at another time developed the same symptoms, which were cured by same treatment. Dr. Ridge reports curing nearly all his cases of tetanus by first giving a large aloes ball, then following it up by squibbs ether in ℥ss doses, three times a day, always placing them in slings immediately. Dr. Goentner has good success with bromide of potash.

Dr. Ridge reported a case of hæmatoma on inside of a cow's leg. Autopsy revealed a large sac filled with coagulated blood, the tumor had several branches of arteries from posterior radial which emptied into the sac.

There were four names proposed for membership, who were instructed to appear before the board of trustees at January meeting. Meeting adjourned.

W. H. Ridge, *Secretary.*

---

The Veterinary Society of the University of Pennsylvania Students was organized October 29th, 1888, "for the purpose of affording "its members opportunity for discussion and investigation of sciences relat-"ing to the diseases of the domesticated animals, their care and breeding, "and all things pertaining to a veterinarian's practice. It will also be the "aim of the society to create a strong band of fellowship between the "graduate and under-graduate members."

The society will also maintain a library for the students.

The officers elected were Leonard Pearson, New York, President; Edgar Tully, Pennsylvania, Vice President; J. M. Eshleman, Treasurer; B. F. Senseman, Secretary; C. F. Oat, Leonard Pearson and A. S. Wheeler, Executive Committee. At the first meeting papers were read by Messrs. E. S. Muir and H. L. Eddy on "*Pharmaceutical Advice* to the Veterinarian," and "The Demand for Veterinarians in the West."

The second meeting of the Society was held November 12th, at which an address was given by Prof. Huidekoper on "Army Veterinary Service."

The history of this branch of army organization in Enropean countries was considered, its beneficial results pointed out, and the pressing need of an efficient service in this country delineated.

A debate on the *question*.

*Resolved*, "That the Veterinarian should have "an infirmary" by Messrs. Baunister, Bathan, Larzelere and Tully, brought out many good points on both sides of the question.

Several interesting cases were reported, among them a complicated case of Pneumonia and Glanders observed by Mr. H. A. Meisner; and a peculiar case of Dyspnœa, observed by Mr. E. M. Wichner, in a horse that worked on a tread-power, in which the trouble was caused by constantly striking the lower part of the neck against the bar that passed in front of the horse.

The following were elected honorary members :—

Profs. Huidekoper, Zuill, Marshall and Harger, Doctors Magill and Lusson.

The society has made a most auspicious beginning and promises to be a great benefit to the students interested.

LEONARD PEARSON, *President.*

